# Uptake of Mesenchymal Stem Cell-Derived Exosomes in Mouse Brain through Intranasal Delivery

**DOI:** 10.2174/0115672018339798240904171503

**Published:** 2024-10-01

**Authors:** Zihe Zhang, Siqi He, Weijie Jiang, Jing Lu, Songbin Liu, Wenjun Xu, Zhi Wang, Fangfang Lu, Qiguo Xiao, Jia Zhang

**Affiliations:** 1 The Second Affiliated Hospital, Hengyang Medical School, University of South China, Hengyang 421001, China;; 2 School of Basic Medicine, Gannan Medical University, Ganzhou 341000, China

**Keywords:** Uptake, mesenchymal stem cells derived exosomes, brain, intranasal delivery

## Abstract

**Introduction:**

Exosomes are nanoscale extracellular vesicles that widely participate in intercellular communication. An increasing number of studies have reported on the neuroprotective effects of stem cell-derived exosomes in brain diseases through various delivery methods. However, only a few reports are available on the delivery and uptake of stem cell-derived exosomes in the brains of mice of different ages.

**Methods:**

PKH-26-labelled mesenchymal stem cell-derived exosomes were collected, and their uptake was investigated in the brains of mice aged 2 weeks, 2 months, and >6 months, 24 hours after intranasal delivery.

**Results:**

No exosomes were distributed in the whole brains of 2-week-old mice after 24 hours of intranasal delivery. However, a small number of exosomes were found in the olfactory bulb, cortex, and hippocampus of 2-month-old mice, with no exosomes observed in the cerebellum. In contrast, a large number of exosomes were ingested in all brain regions, including the olfactory bulb, cortex, hippocampus, and cerebellum, of >6-month-old mice.

**Conclusion:**

Exosomes can enter the brains of adult mice through intranasal administration, but there are differences in the uptake rate among mice of different ages. These findings provide a theoretical basis for the future clinical administration of exosomes for treating brain disorders.

## 1 INTRODUCTION

Exosomes are extracellular vesicles (EVs) with a diameter ranging from 30 to 150 nm, secreted by nearly all cell types [1-4]. These vesicles have garnered significant attention as crucial mediators of intercellular communication, playing key roles in regulating cell proliferation, migration, organization, and phenotype during various physiological and pathological processes, including development, maintenance, injury, disease, and aging [5-10]. Structurally, exosomes exhibit a distinctive cup-shaped morphology with a double-layered membrane that carries several biomarkers, such as CD9, CD63, and CD81, on its surface [11, 12]. Composed of proteins, lipids, amino acids, and nucleic acids, exosomes typically contain molecules like TSG101, HSP70, HSP90, ALIX, mRNA, miRNA, and lnRNA [13-17]. Increasing evidence suggests that stem cell-derived exosomes have the potential to effectively repair neuronal damage associated with Central Nervous System (CNS) diseases, such as Alzheimer’s disease [18-22], Parkinson’s disease [23-28], spinal cord injury [29-33], and retinal disorders [34-40]. Despite these promising findings, reports on the delivery and uptake of stem cell-derived exosomes in the brain remain scarce.

Studies have demonstrated that exosomes can be absorbed by cells through multiple pathways, including receptor-mediated endocytosis, direct binding, clathrin-coated pits, lipid rafts, phagocytosis, caveolae, macropinocytosis, and direct fusion [15, 41, 42]. For instance, in a retinal ischemia-reperfusion model, stem cell-derived exosomes were taken up by retinal cells *via* the caveolar endocytic pathway following intravitreous injection, leading to the protection of retinal neurons and the promotion of visual function recovery [43]. These findings suggest that direct topical application of exosomes to lesion sites can effectively enhance their uptake by target organs or cells. However, the brain presents a unique challenge for exosome delivery due to its complex structure and the difficulty of directly contacting and delivering exosomes to specific lesion areas. In previous studies involving brain disease models in mice, exosomes were predominantly administered through tail vein and lateral ventricle injections [44-48]. Nevertheless, both methods pose significant challenges for clinical application. Tail vein injections are hindered by the blood-brain barrier (BBB), which restricts the delivery of most drugs to the brain [49-51]. Although exosomes can cross the BBB, their rapid uptake and subsequent metabolism by liver and spleen cells often prevent them from fully reaching the brain. On the other hand, lateral ventricle injections require craniotomy, which can cause brain damage.

Intranasal administration, however, offers a non-invasive alternative that targets the brain directly, bypassing systemic exposure and achieving high brain bioavailability with low associated doses, thereby minimizing adverse effects [52-57]. This route has proven effective in delivering drugs to the brain for treating CNS disorders, such as Alzheimer’s disease, Parkinson’s disease, epilepsy, and psychiatric conditions [58-62]. Despite its potential, the mechanisms underlying the transport of exosomes *via* intranasal administration remain unclear, and detailed studies on the uptake of exosomes in various brain regions are lacking.

Research has shown that intranasal administration may be performed through two pathways: intracellular and extracellular. The intracellular pathway involves the endocytosis of olfactory cells, which then transport the exosomes along axons to synaptic gaps in the olfactory bulb, where they are released and subsequently distributed to other brain regions. In the extracellular pathway, exosomes pass through the paraepithelial space of the nasal epithelium, reach the brain’s subarachnoid space *via* the perineural space, and are directly transported to the cerebrospinal fluid [63-67]. Understanding whether exosomes follow these pathways during intranasal administration is crucial for optimizing their therapeutic potential.

In this study, we investigated the uptake of stem cell-derived exosomes in the brains of mice aged 2 weeks, 2 months, and >6 months following intranasal administration. Specifically, we examined the distribution of exosomes in the olfactory bulb, cortex, hippocampus, and cerebellum. Our findings revealed that no exosomes were detected in the brains of 2-week-old mice. However, in 2-month-old mice, exosomes were present in the olfactory bulb, cortex, and hippocampus, with no detectable uptake in the cerebellum. In >6-month-old mice, exosomes were widely distributed across all examined brain regions, including the olfactory bulb, cortex, hippocampus, and cerebellum. These results indicated that intranasally administered exosomes can enter the brains of adult mice, but the uptake rate varies significantly with age. This study provides a theoretical foundation for the future clinical application of exosomes in treating brain disorders.

## 2 MATERIAL AND METHODS

### 2.1 Animals

For this study, 2-week-, 2-month- and >6-month-old C57BL/6J mice were purchased from Guangdong Medical Experimental Animal Center (Guangzhou, China). The mice were kept in a controlled animal facility (23°C±2°C, 60%±5% relative humidity) with a standard 12-hour light/12-hour dark cycle and provided access to food and water. Four to five C57BL/6J mice were stored per cage. All animal experiments were conducted following guidelines developed by the University of South China’s Animal Care and Use Committee, which approved all the protocols used in this study.

### 2.2 Acquisition of Exosomes and PKH26 Staining

The acquisition of exosomes PKH26 staining was conducted as previously described [34]. Briefly, human umbilical cord mesenchymal stem cells isolated from huamn umbilical cord tissue were inoculated with 1×10^6^ cells /mL in a 10-cm dish and cultured in DMEMF/12 containing 10% foetal bovine serum. When the cell confluency was approximately 80%–90%, the medium was changed, and the cell supernatant was collected for 24–48 hours. The exosomes were harvested using ultra-high-speed centrifugation. First, large cell debris was removed by centrifugation at 2,500g for 30 min at 4°C, and the supernatant was retained to remove small debris by centrifugation at 10,000g for 45 min at 4°C. Finally, the supernatant was collected by centrifugation at 100,000g for 90 min and discarded to obtain EVs. The experimental procedures were performed according to the manufacturer’s instructions provided in the PKH26 Red Fluorescent Cell Linker Kit (Cat.PKH26) to label exosomes.

### 2.3 Nasal Administration

PKH26-labelled exosomes were re-suspended in sterile PBS solution at a concentration of 6 μg/μL (30 μg of the corresponding vesicle protein, approximately 15×10^9^ vesicles). The mice were anaesthetised with isoflurane and carefully administered PBS solution (control groups) or exosomes intranasally at a volume of 5 μL per mouse. This dosing protocol was consistent across all age groups, ensuring that each mouse received a total of 30 μg of exosomes. After 24 hours, the mice were anesthetized again with tribromoethanol, followed by fixation with 4% PFA for immunofluorescence staining. The 24-hour post-administration period was selected based on preliminary data and prior literature to capture the distribution and retention of exosomes in the brain [68, 69].

### 2.4 Immunofluorescence Staining

The PFA-fixed brain tissue was subjected to gradient dehydration: 30% sucrose dehydration for 24 hours on the first day and 35% sucrose dehydration on the second day. Subsequently, an optimal cutting temperature compound was used for embedding, and the tissues were stored at −80°C. Next, a 25-μm-sized coronal brain tissue section was used to observe the uptake of exosomes in the CA1, CA3, and DG regions of the medial hippocampus, olfactory bulb, cortex, and cerebellum. After incubation with a blocking solution (30% bovine serum albumin in PBS and 0.3% Triton X-100 (Sigma-Aldrich)) for 100 min, Iba1 (019-19741, Wako), Map2 (8707s, CST), and β3-Tubulin (5568s, CST) were incubated at 4°C overnight and with the corresponding fluorescent secondary antibody for 60 min the next day. The samples were collected using a confocal laser scanning microscope (Zeiss). Brain tissues that were not adequately preserved or processed for analysis were excluded from the data set.

### 2.5 Experiment Design and Data Analysis

For the analysis of exosome uptake in different brain regions (hippocampus, olfactory bulb, cortex, and cerebellum), 12 mice were used for each experiment. Each mouse was assigned a random number generated using a computerized random number generator, and then these numbers were randomly sorted to allocate mice to either the PBS control group or one of the three different age treatment groups (2 weeks, 2 months, and >6 months), with each age group consisting of 3 mice. The choice of using 3 mice per age group aligns with common practices in preclinical studies to provide initial insights while minimizing animal use. This sample size was deemed sufficient to observe significant differences in exosome uptake across different brain regions and age groups, given the exploratory nature of the study.

No quantitative statistical analyses were performed for data analysis in this study. The research focused on qualitative analysis to observe the distribution and uptake of mesenchymal stem cell-derived exosomes in different brain regions of mice across various age groups. The qualitative data were assessed visually through microscopy and imaging techniques without the use of statistical software or formal statistical tests. Researchers conducting the exosome administration and subsequent brain tissue analysis were blinded to the group assignments to reduce observer bias.

## 3 RESULTS

### 3.1 The Uptake of Exosomes was Effective in the Olfactory Bulb of Adult Mice After 24 Hours of Intranasal Delivery

**Fig. (1):**
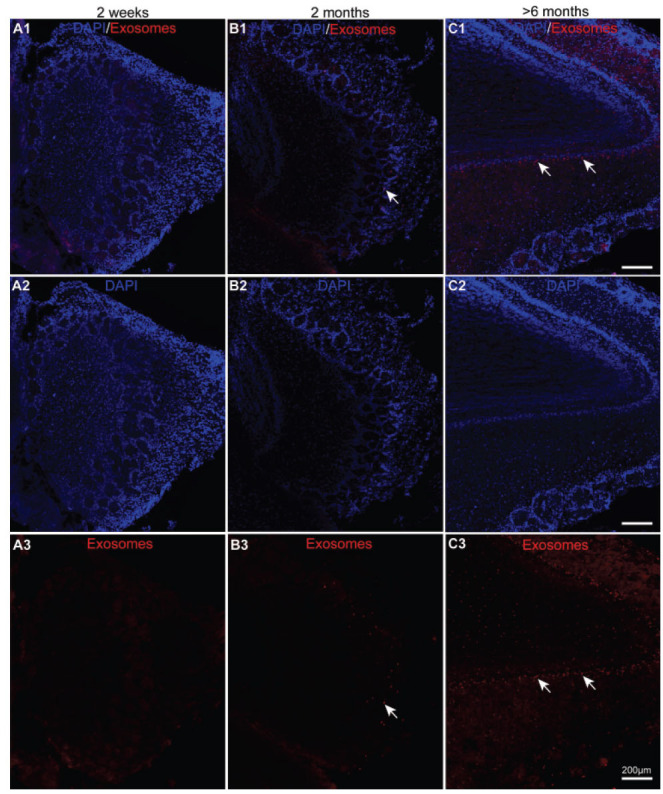
The uptake of exosomes in the olfactory bulb of the three different-aged mouse groups after 24 hours of intranasal delivery. (**A1-C3**) Confocal fluorescence microscopy was used to capture the images of the PKH26 (red)-labelled exosomes and DAPI (blue)-stained olfactory bulbs of the 2-week-, 2-month-, and >6-month-old mice.

To identify the two pathways for the intranasal administration of exosomes, 2-week-, 2-month- and >6-month-old mice were administrated 30 μg of PKH-26-labelled exosomes or PBS as control. The endocytosis of the olfactory bulb was the first target cerebral area for the intracellular and extracellular pathways. Therefore, we first observed the uptake of exosomes in the olfactory bulb. As shown, almost no exosomes were ingested in the olfactory neurons at 2 weeks after 24 hours of intranasal administration (Fig. **1A**, *n=*3). Contrary to that observed in the 2-week-old mice, exosomes were observed in the olfactory bulb (Figs. **1B** and **C**, white arrows) in the 2-month and >6-month-old mice. In addition, the uptake rate seemed to be higher in the olfactory bulb of the older mice (Figs. **1A3**, **B3**, and **C3**, *n=*3). Moreover, there were no visible PKH26-positive signals in the control groups from three different ages (Supplementary Fig. **1**, *n=*3). Overall, the uptake of exosomes through intranasal delivery was effective in the olfactory bulb of the adult mice but not the juvenile mice.

### 3.2 The Exosomes Were Ingested Effectively in the Hippocampus of the Older Mice After 24 Hours of Intranasal Delivery

**Fig. (2):**
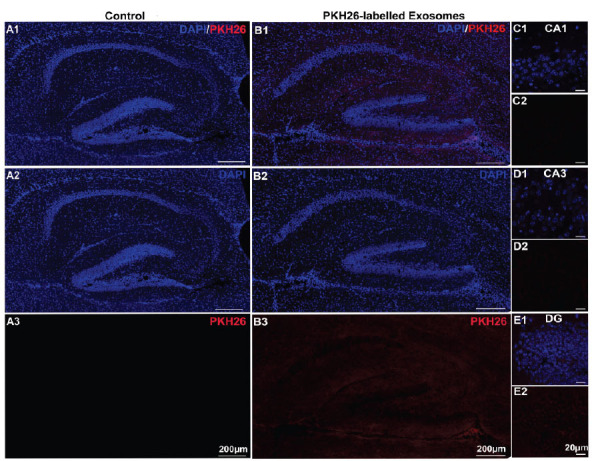
The uptake of exosomes in the hippocampus of the 2-week-old mice after 24 hours of intranasal delivery. (**A1**-**B3**) Images of PKH26-labelled exosomes (red) and DAPI (blue) staining in the hippocampus of the 2-week-old mice treated with intranasal administration of (**A1**-**A3**) PBS or (**B1**-**B3**) PKH26-labelled exosomes. (**C**-**E**) The PKH26-labelled exosomes (red) and DAPI (blue) staining in the CA1, CA3, and DG regions.

**Fig. (3):**
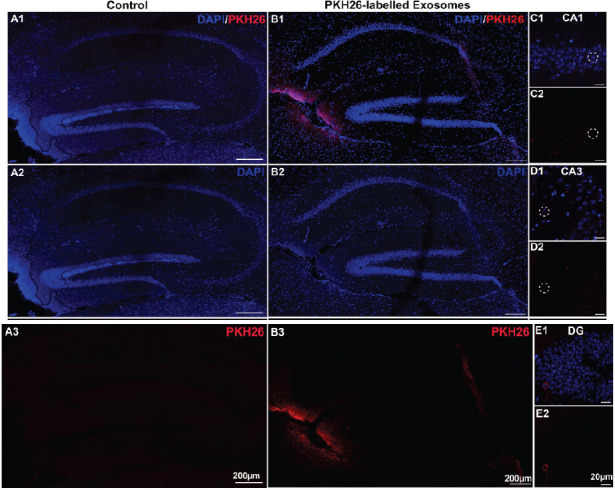
The uptake of exosomes in the hippocampus of the 2-month-old mice after 24 hours of intranasal delivery. (**A1**-**B3**) Images of PKH26-labelled exosomes (red) and DAPI (blue) staining in the hippocampus of the 2-month-old mice treated with intranasal administration of (**A1**-**A3**) PBS or (**B1**-**B3**) PKH26-labelled exosomes. (**C-E**) The PKH26-labelled exosomes (red) and DAPI (blue) staining in the CA1, CA3, and DG regions.

**Fig. (4):**
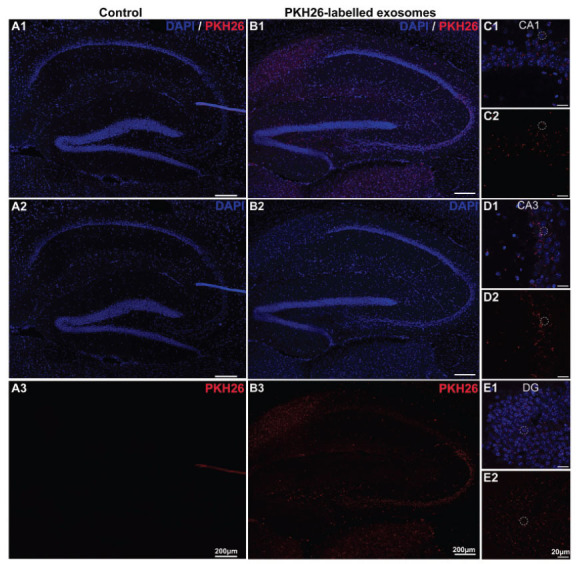
The uptake of exosomes in the hippocampus of the >6-month-old mice after 24 hours of intranasal delivery. (**A1**-**B3**) Images of PKH26-labelled exosomes (red) and DAPI (blue) staining in the hippocampus of the >6-month-old mice treated with intranasal administration of (**A1**-**A3**) PBS or (**B1**-**B3**) PKH26-labelled exosomes. (**C-E**) The PKH26-labelled exosomes (red) and DAPI (blue) staining in the CA1, CA3, and DG regions.

To explore the transportation of the exosomes in the other brain regions after that in the olfactory bulb, the uptake of PKH26-labelled exosomes and PBS control in the hippocampus of the three different-aged mouse groups was observed. As shown in Fig. (**2-4**), only minimal PKH26-labelled exosomes were observed in the hippocampus of the 2-week-old mice. However, it is important to note that while the exosome presence was sparse, some exosomes were distributed in the olfactory nerve of 2-week-old mice (Figs. **2B**-**E**, *n=*3), suggesting a potential for limited distribution within certain brain regions. In contrast, a small number of PKH26-labelled exosomes were found in the hippocampus of the 2-month-old mice (Figs. **3B**-**E**, *n=*3). This suggests a moderate level of exosome uptake at this age. In particular, the PKH26-labelled exosomes were clearly observed in the CA1 and CA3 regions (Figs. **3C**-**D**, *n=*3). To our interest, a large number of exosomes were absorbed in the CA1, CA3, DG, and other hippocampal regions in the >6-month-old mice through intranasal delivery (Figs. **4B-E**, *n=*3), indicating a much higher efficiency of exosome uptake through intranasal delivery at this age. Additionally, no PKH26-positive signals were observed in the hippocampus from all the control groups (Figs. **2**, **3**, and **4A**). These results suggested that the exosomes seemed to be ingested effectively through intranasal delivery in the hippocampus of the older mice.

### 3.3 The Exosomes were Ingested Effectively in the Cortex of the Older Mice After 24 Hours of Intranasal Delivery

**Fig. (5):**
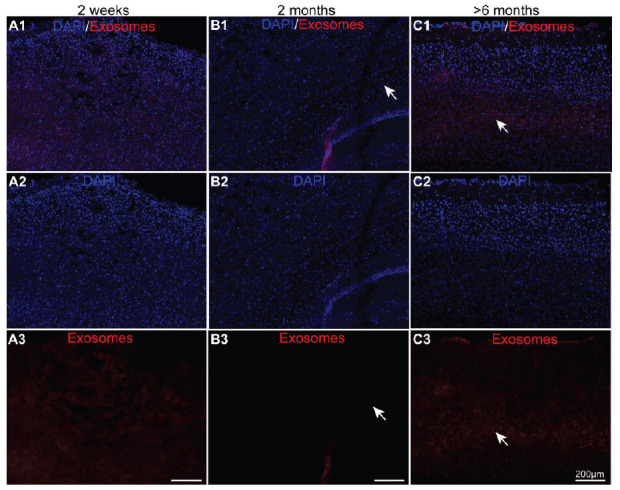
The uptake of exosomes in the cortex of the three different-aged mice groups after 24 hours of intranasal delivery. (**A1-C3**) Confocal fluorescence microscopy was used to capture the images of PKH26 (red)-labelled exosomes and DAPI (blue) staining in the cortexes of the 2-week-, 2-month-, and >6-month-old mice.

To explore the transportation of exosomes in the other brain regions, we investigated the uptake of PKH26-labelled exosomes in the cortex of the three different-aged mice groups. Similarly, almost no PKH26-labelled exosomes were observed in the cortex of the 2-week-old mice (Fig. **5A**, *n=*3). However, a small number of PKH26-labelled exosomes were found in the cortex of the 2-month-old mice (Fig. **5B**, *n=*3). As shown in Fig. (**5C**), large amounts of exosomes were observed in the cortex of the >6-month-old mice. Besides, no PKH26-positive signals were observed in the cortex from three different-aged control groups (Supplementary Fig. **2**, *n=*3). These results also suggested that exosomes seemed to be ingested effectively in the cortex of older mice through intranasal delivery.

### 3.4 The Uptake of Exosomes was Only Effective in the Cerebellum of the Old Aged Mice After 24 Hours of Intranasal Delivery

**Fig. (6):**
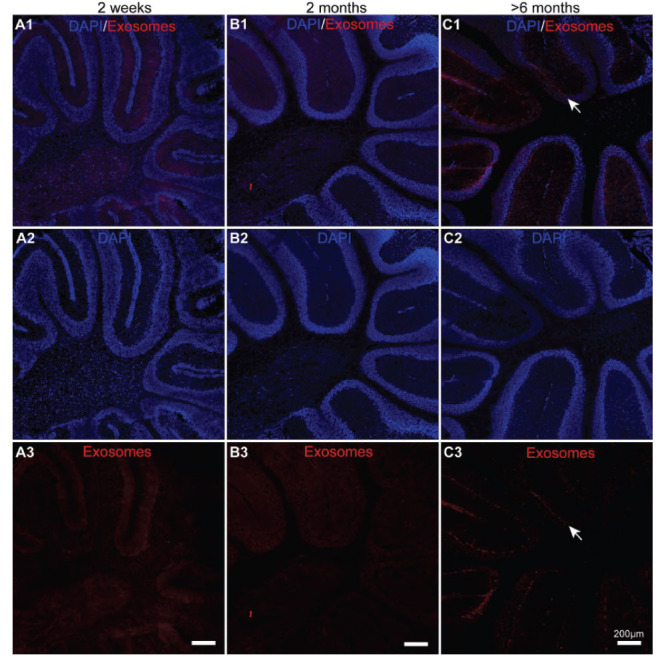
The uptake of exosomes in the cerebellum of the three different-aged mice groups after 24 hours of intranasal delivery. (**A1**-**C3**) Confocal fluorescence microscopy was used to capture the images of PKH26 (red)-labelled exosomes and DAPI (blue) staining in the cerebellum of the 2-week-, 2-month-, and >6-month-old mice.

To explore the transportation of exosomes in the distant brain regions, the uptake of PKH26-labelled exosomes in the cerebellum of the three different-aged mouse groups was investigated. To our interest, nearly no exosomes were found after 24 hours of intranasal delivery in the cerebellum of the 2-week-old or 2-month-old mice (Figs. **6A-B**, *n=*3). Unlike in these two mouse groups, in the >6-month-old mice, large amounts of exosomes were discovered in the cerebellum (Fig. **6C**, *n=*3). Moreover, no PKH26-positive signals were observed in the cerebellum from three different-aged control groups (Supplementary Fig. **3**, *n=*3). Overall, the uptake of exosomes was only effective in the cerebellum of the >6-month-old mice after 24 hours of intranasal delivery.

### 3.5 The Endocytosis of Exosomes was Absorbed by Microglia and Neurons in the Hippocampus of the >6-Month-old Mice After 24 Hours of Intranasal Delivery

**Fig. (7):**
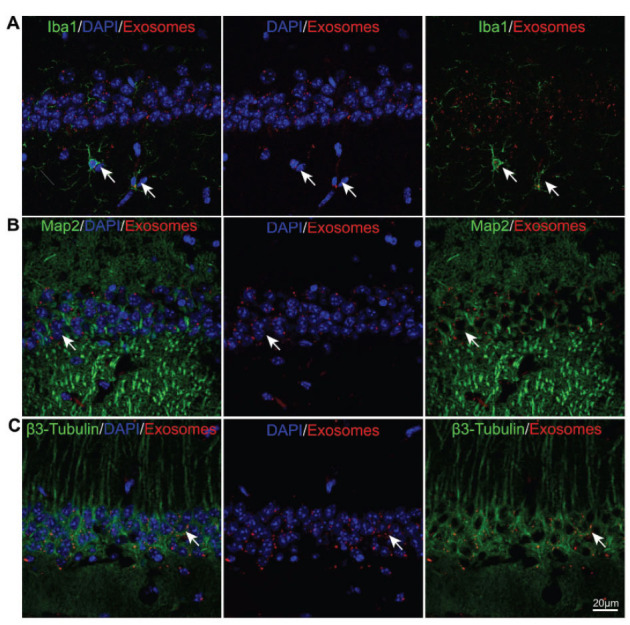
The microglia and neurons ingest the exosomes in the CA1 region in the hippocampus of the >6-month-old mice. (**A-C**) Images of immunofluorescence staining with an antibody against Iba1 (green), Map2 (green), β3-Tubulin (green), and DAPI (blue) and the co-labelling of brain slices with exosomes (red) in the >6-month-old mice.

To further identify the cell-targeting specificity of exosomes, the uptake distribution of the exosomes labelled with the fluorescent dye PKH26 in the hippocampus was observed. After 24 hours of the intranasal delivery of the exosomes, the brain tissue was fixed and stained with Iba1, Map2, and β3-Tubulin (Fig. **7**, *n=*3). The results showed that Iba-1 microglia were PKH26^+^ (Fig. **7A**, *n=*3), indicating that the injected exosomes were absorbed by microglia. Simultaneously, Map2 and β3-Tubulin neurons also manifested as PKH26^+^ (Figs. **7B** and **C**, *n=*3), showing that the injected exosomes were absorbed by neuronal cells.

## 4 DISCUSSION

The potential of intranasal delivery as a route for brain-targeted therapies has been increasingly recognized, with early studies demonstrating the uptake of exosomes labelled with dyes or PKH26 fluorescence in the mouse brain as early as 2011. These studies highlighted the rapid endocytosis of exosomes within 30 minutes post-administration [70]. Following this, numerous investigations have confirmed the efficient uptake of exosomes through intranasal administration across various contexts, with uptake times ranging from 6 to 24 hours [71-75]. However, many of these studies have not detailed the distribution of exosomes across different brain regions or considered the impact of age on exosome uptake, which is critical for optimizing therapeutic outcomes. In our study, we specifically aimed to elucidate the long-term retention and distribution of exosomes in different brain regions, including the olfactory bulb, hippocampus, cortex, and cerebellum, across different-aged mouse groups under normal physiological conditions.

Previous research, such as that by Zhai *et al*., reported the uptake of exosomes derived from HEK293T cells loaded with BDNF after 18 hours of intranasal delivery, with significant distribution observed in the olfactory bulb, thalamus, and cerebral cortex [51]. However, this study focused solely on 7-week-old mice, leaving gaps in our understanding of exosome distribution in both younger and older mice. Other studies using engineered exosomes in models of stroke and Parkinson's disease also did not provide data on exosome distribution in 10-week-old mice or during early post-administration phases [76, 77]. For instance, the highest uptake in a Parkinson's model was noted at 6 hours [77], contrasting with another study that observed significant exosome presence in injured brain areas 24 hours post-administration in a traumatic brain injury model [69]. These findings prompt further questions about how exosomes migrate to injured areas and the differences in their uptake under pathological conditions compared to normal physiological states.

Our findings indicate that after 24 hours of intranasal delivery, exosome distribution in 2-week-old mice was minimal across all brain regions examined. However, as the mice aged, there was a notable increase in exosome uptake. Specifically, 2-month-old mice showed some exosome distribution in the olfactory bulb, with lesser amounts in the hippocampus and cortex and almost none in the cerebellum. In contrast, in >6-month-old mice, exosomes were widely distributed across nearly all brain regions, suggesting that age significantly impacts exosome uptake in the brain. Several factors may contribute to these age-related differences. Firstly, the permeability of the nasal mucosa may be higher in older mice compared to younger ones, which could limit the amount of exosomes reaching the brain in younger mice, making them more challenging to detect. Secondly, the metabolic rate of brain cells might be higher in younger mice, leading to faster metabolism of ingested exosomes, thereby reducing their detectability after 24 hours. Furthermore, the 24-hour observation period chosen in this study might have been too long for younger mice, where shorter intervals such as 8 or 16 hours could potentially reveal more pronounced uptake patterns.

Given these considerations, the conclusions drawn from our study, specifically that “2-week-old mice exhibited minimal exosome uptake”, were based on the specific conditions and time points we utilized. Future studies should explore shorter observation periods to fully understand the dynamics of exosome uptake across different age groups. Moreover, further research is needed to elucidate the specific factors influencing these results and to optimize intranasal exosome delivery for therapeutic applications.

## CONCLUSION

Our study demonstrated that mesenchymal stem cell-derived exosomes can effectively enter the brains of adult mice through intranasal administration, with significant variations in uptake rates across different age groups. Specifically, 2-week-old mice exhibited minimal exosome uptake, 2-month-old mice showed moderate uptake, and >6-month-old mice displayed extensive distribution of exosomes throughout various brain regions. These age-related differences underscore the critical roles of nasal mucosa permeability and metabolic activity in influencing exosome uptake.

Our findings provide valuable baseline data that can serve as a foundation for future studies involving disease models, as well as a basis for optimizing intranasal exosome delivery in clinical settings for treating brain disorders. Further research is necessary to elucidate the mechanisms underlying exosome uptake, particularly in younger animals, and to explore the potential of shorter observation periods that may reveal more dynamic uptake patterns. Understanding these mechanisms will be crucial for the development of effective exosome-based therapies for brain disorders, both under normal physiological conditions and in the presence of pathology.

## STUDY LIMITATIONS

The following are the limitations of this study.

### Single-dose and Single-timepoint Study Design

We used a single intranasal dose (30 µg) of labeled exosomes and evaluated brain distribution at a single time point (24 hours post-administration), which restricted our ability to assess dose-response effects and biodistribution dynamics over time.

### Absence of Disease Models

The study was performed exclusively on healthy mice. Therefore, it remains uncertain how effective the intranasal delivery of exosomes would be under pathological conditions, such as neuroinflammation or neurodegeneration.

### No Assessment of Peripheral Biodistribution or Safety Profile

The biodistribution of exosomes in peripheral organs (*e.g*., liver, lung, spleen, *etc*.) was not evaluated, nor were any toxicity or immunogenicity studies conducted. These aspects are important for translational considerations.

### Lack of Mechanistic and Functional Studies

While we demonstrated exosome presence in various brain regions, we did not investigate the specific uptake pathways (e.g., olfactory neuronal vs. extracellular routes) or the biological consequences of exosome internalization in recipient brain cells.

## Data Availability

The datasets used and analysed during the current study are included within the article and its supplementary files.

## LIST OF ABBREVIATIONS

ALIX: ALG-2-interacting Protein X
BBB: Blood-Brain Barrier
CA1/CA3/DG: Subregions of the Hippocampus
CNS: Central Nervous System
DMEM/F12: Dulbecco's Modified Eagle's Medium/Nutrient Mixture F-12
EVs: Extracellular Vesicles
HSP70: Heat Shock Protein 70
HSP90: Heat Shock Protein 90
lnRNA: Long Non-coding Ribonucleic Acid
miRNA: Micro Ribonucleic Acid
mRNA: Messenger Ribonucleic Acid
PBS: Phosphate-Buffered Saline
PFA: Paraformaldehyde
PKH-26: A Fluorescent Cell Linker Dye
TSG101: Tumor Susceptibility Gene 101 Protein
