# Supplementary material for: Uptake of Mesenchymal Stem Cell-Derived Exosomes in Mouse Brain through Intranasal Delivery
Source: Curr Drug Deliv. 2024 Oct 1;22(8):1112–24. doi: 10.2174/0115672018339798240904171503 (PMC12645106; doi:10.2174/0115672018339798240904171503)
Supplement: Supplementary file 1 [file CDD-22-8-1112_SD1.pdf]

SUPPLEMENTARY MATERIAL

Uptake of Mesenchymal Stem Cell-Derived Exosomes in Mouse Brain through Intranasal Delivery

Zihe Zhang[#], Siqi He[1,#], Weijie Jiang[1], Jing Lu[1], Songbin Liu[1,2], Wenjun Xu[1,2], Zhi Wang[1], Fangfang Lu[1,*], Qiguo Xiao[1,*] and Jia Zhang[1,*]

[1]*The Second Affiliated Hospital, Hengyang Medical School, University of South China, Hengyang 421001, China;* [2]*School of Basic Medicine, Gannan Medical University, Ganzhou 341000, China*

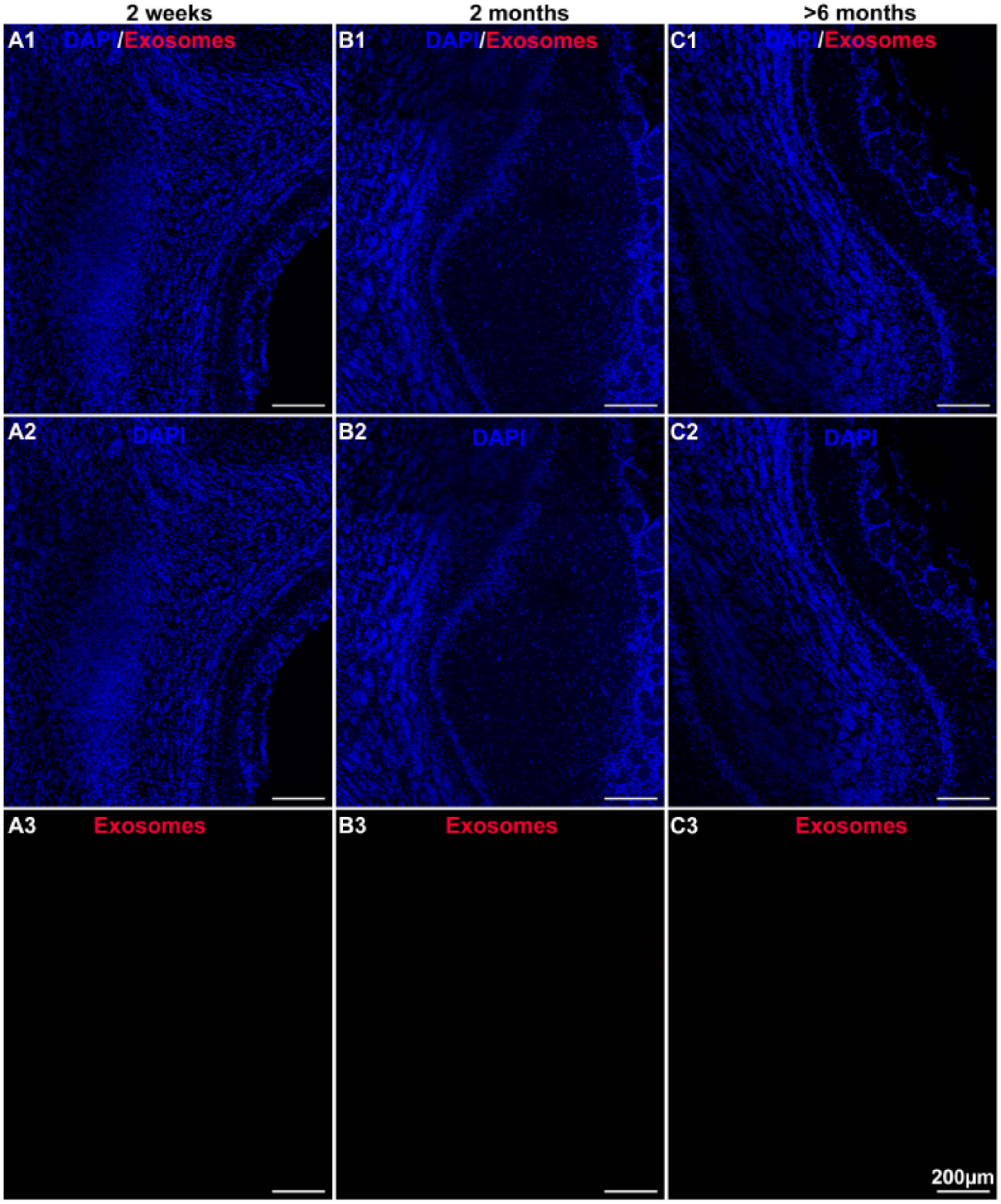

Supplementary Fig. (1). The uptake of exosomes in the olfactory bulb of the three different-aged mouse groups after 24 hours of intranasal delivery of PBS. (A-C) Confocal fluorescence microscopy was used to capture the images of the PKH26 (red) and DAPI (blue) staining in the olfactory bulbs of the 2-week-, 2-month-, and >6-month-old mice.

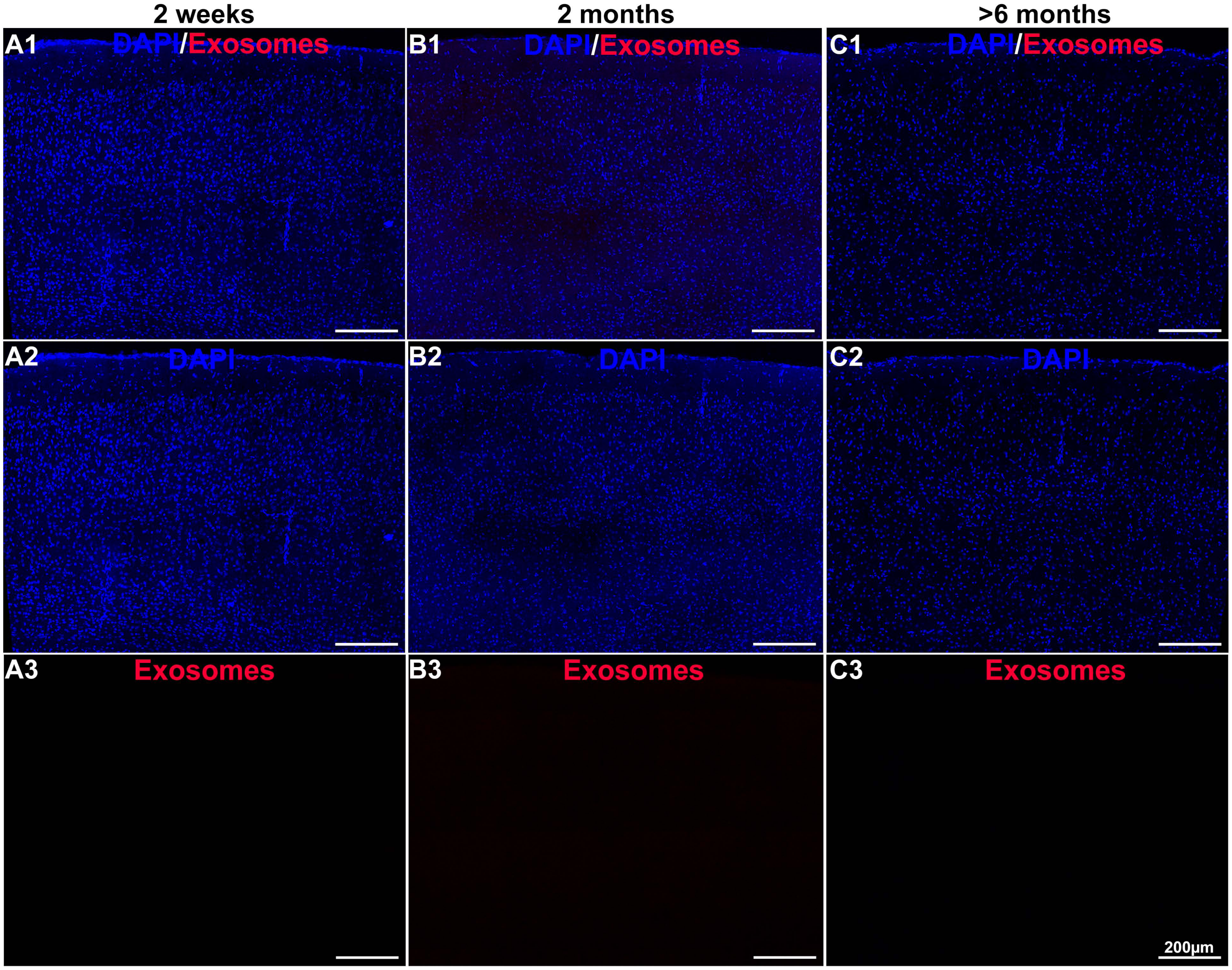

Supplementary Fig. (2). The uptake of exosomes in the cortex of the three different-aged mice groups after 24 hours of intranasal delivery of PBS. (A-C) Confocal fluorescence microscopy was used to capture the images of PKH26 (red) and DAPI (blue) staining in the cortexes of the 2-week-, 2-month-, and >6-month-old mice.

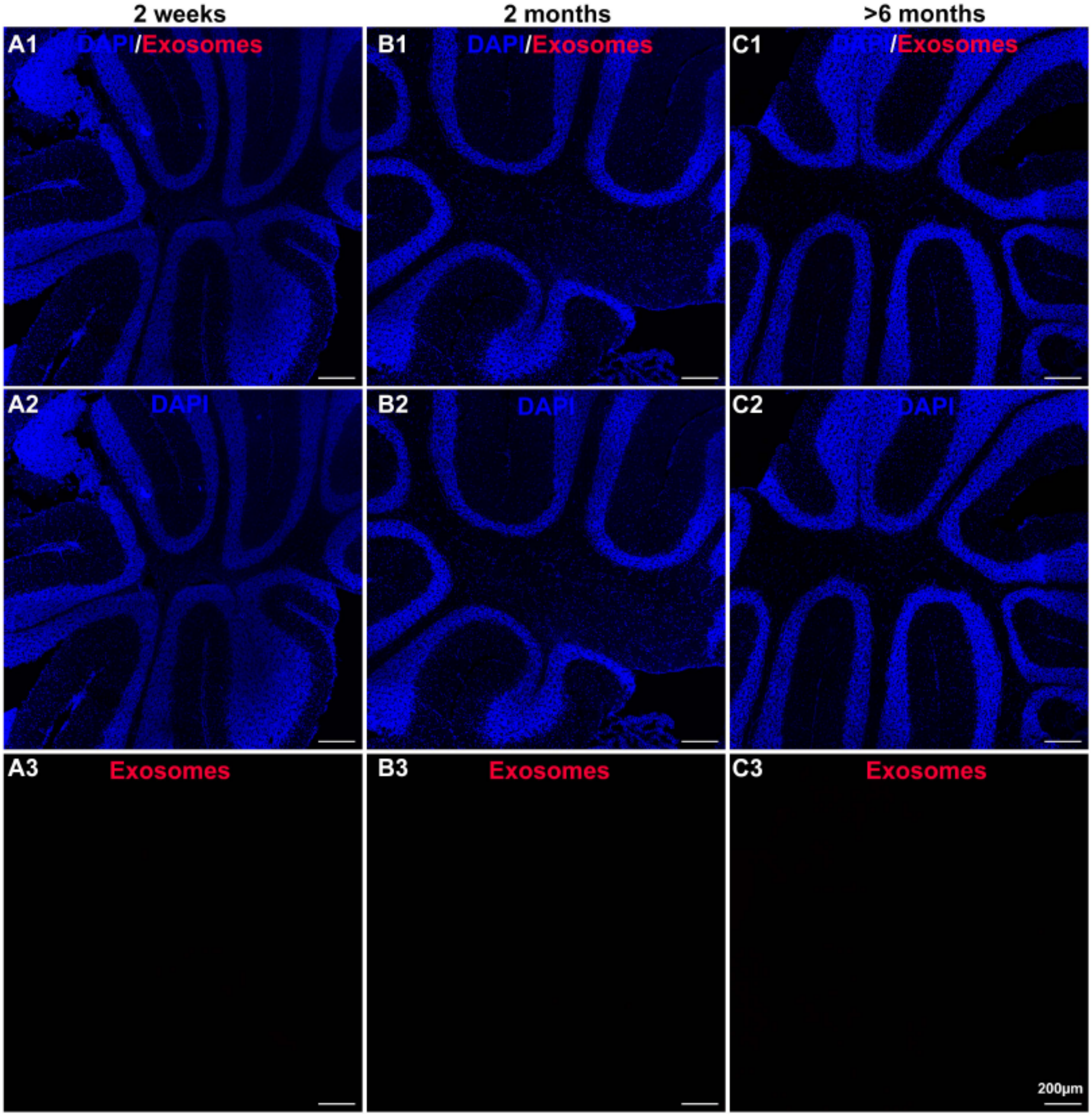

Supplementary Fig. (3). The uptake of exosomes in the cerebellum of the three different-aged mice groups after 24 hours of intranasal delivery of PBS. (A-C) Confocal fluorescence microscopy was used to capture the images of PKH26 (red) and DAPI (blue) staining in the cerebellum of the 2-week-, 2-month-, and >6-month-old mice.
